# Effects of *CYP3A5* Genotypes on Thrombocytopenia in Liver Transplantation Patients Treated with Tacrolimus

**DOI:** 10.3390/biomedicines11113088

**Published:** 2023-11-17

**Authors:** Zhe Guo, Qi Chen, Juan Liu, Shan Li, He Wang, Rui Tang, Zhenyu Zhang

**Affiliations:** 1 Department of Liver Critical Care Medicine, Tsinghua Changgung Hospital, School of Clinical Medicine, Tsinghua University, Beijing 102218, China; gza01482@btch.edu.cn (Z.G.); lsa00856@btch.edu.cn (S.L.); wha02539@btch.edu.cn (H.W.); 2 Department of Geriatric, Tsinghua Changgung Hospital, School of Clinical Medicine, Tsinghua University, Beijing 102218, China; cqa03755@btch.edu.cn; 3 Hepatobiliary Pancreatic Center, Tsinghua Changgung Hospital, School of Clinical Medicine, Tsinghua University, Beijing 102218, China; lja02720@btch.edu.cn (J.L.); tangrui_hs@163.com (R.T.)

**Keywords:** CYP3A5, deceased-donor liver transplantation (DDLT), tacrolimus, thrombocytopenia

## Abstract

Background: Thrombocytopenia is a complication after liver transplantation. This study’s aims were to evaluate the role of *CYP3A5* genotypes on tacrolimus-induced thrombocytopenia after orthotopic liver transplantation. Methods: In this retrospective case–control study, data from 100 patients who underwent deceased-donor liver transplantation (DDLT) were divided into CYP3A5*3 genotype (donor/recipient) tacrolimus fast- (A*/A*, *n* = 22), intermediate- (A*/GG, *n* = 20; GG/A*, *n* = 31) and slow-metabolizer (GG/GG, *n* = 27) groups. Platelet count changes and prognosis for 180 days after surgery were compared. Results: Platelet counts declined significantly after DDLT, especially on postoperative day (POD) 3, and continued at low levels for a week thereafter in all groups. In the GG/GG group, platelet counts on POD3 (50.29 ± 5.44 × 10^9^/L) were the lowest among the groups (A*/A*, 71.00 ± 6.22 × 10^9^/L; A*/GG, 57.95 ± 6.21 × 10^9^/L; GG/A*, 75.90 ± 5.56 × 10^9^/L) (*p* = 0.006). Compared with the A*/A* genotype, tacrolimus nadir levels were significantly higher in GG/GG genotype patients, who also exhibited a higher incidence of hemorrhage (22.2%, *p* = 0.011). A combination of a nadir blood concentration of tacrolimus ≥ 4.74 ng/mL and spleen size ≥ 165.5 mm was a risk factor for increased thrombocytopenia after DDLT on POD3, with an AUC of 0.735 (sensitivity, 77.2%; specificity, 41.7%). Conclusions: A high blood concentration of tacrolimus after the early stage of DDLT is a major risk factor for hemorrhage. For the *CYP3A5* genotype (GG/GG), controlling the blood concentration of tacrolimus below the target concentration until POD3 can avoid thrombocytopenia-related complications.

## 1. Introduction

Orthotopic liver transplantation (OLT) is an effective treatment for end-stage liver disease and hepatocellular carcinoma within the Milan criteria [1]. Promoting the early regeneration of a graft and avoiding ischemia-reperfusion injury are important factors in improving the prognosis of OLT patients [2]. Platelets promote tissue repair and regeneration by secreting various growth factors and serotonin, and their roles in liver regeneration after partial hepatectomy have been attributed to effects on hepatocytes as well as on hepatic endothelial and Kupffer cells [3].

Platelet count reductions that occur on postoperative days (PODs) 3–5 are correlated with early graft dysfunction [4] and biliary anastomotic stricture [5], which is one of the risk factors for predicting early complications and even increased grade III b/IV complications [6]. Hemodilution, platelet sequestration in the liver graft or spleen, and especially immunosuppressive medications have been proposed to affect platelet counts after liver transplantations [3]. Tacrolimus is a calcineurin inhibitor and is typically used in combination with other immunosuppressive medication. Tacrolimus is commonly used to inhibit the rejection of the graft in solid organ transplantations. Its inhibitory effects on T cell activation, with an immunosuppressive activity 10–100-fold higher than that of cyclosporine, results in a therapeutic trough blood concentration around 20-fold lower than cyclosporine [7]. This leads to a narrow therapeutic index since underexposure might increase the risk of rejection, while overexposure may increase the risk of toxicity [8,9].

The dosage and administration of tacrolimus are typically individualized based on a patient’s specific medical condition, response to treatment and other factors [10]. To maximize efficacy combined with safety, various methods of therapeutic drug monitoring (TDM) have been developed to inform adjustments to tacrolimus doses including the tacrolimus target trough concentration determination and red blood cell count, as well as the plasma population, pharmacokinetics and other prediction models [11,12,13,14]. However, because of large interpatient pharmacokinetic variability, efficient tacrolimus monitoring still remains challenging [8,15,16,17]. As a recent approach to overcome inter-individual variations in the pharmacokinetic and pharmacodynamics profiles of tacrolimus, it has been proposed to include polymorphism detections for personalized treatments [18,19].

The P450 family 3, subfamily A polypeptide 5 (*CYP3A5*), is the main metabolic enzyme of tacrolimus, and its mutation at the CYP3A5*3 locus 6986 (adenine (A) > guanine (G) mutation in intron 3) leads to the premature termination of DNA transcription, resulting in the translation of non-functional proteins. As a result, *CYP3A5* GG genotype carriers cannot express the CYP3A5 protein with enzyme activity, though 80–85% of the white population is homozygous for this mutation [18]. In a previous study, *CYP3A5* mutations were proposed to be useful for the optimization of cyclosporine A dosing in patients requiring renal allografts since *CYP3A5*- and *CYP3AP1*-linked mutations were found to correlate with necessary cyclosporine doses at 3 and 6 months post transplantation [20]. In addition, another study noted that *CYP3A5* mutant genotypes were related to anti-tuberculosis-drug-induced hepatotoxicity (ADIH) development in tuberculosis patients receiving anti-tuberculosis chemotherapy [21].

The purpose of the present study was to determine correlations between tacrolimus serum concentration differences caused by diverse *CYP3A5* genotypes and the occurrence of thrombocytopenia in the early stages after deceased-donor liver transplantation (DDLT).

## 2. Materials and Methods

### 2.1. Patients

In this retrospective case–controlled study, data from 131 patients who underwent DDLT in our hospital from January 2020 to January 2021 were collected and analyzed. The inclusion criteria were patients aged ≥ 18 years; a tacrolimus + mycophenolate mofetil (MMF) + methylprednisolone combined anti-rejection regimen given within 24 h after surgery; and a postoperative prophylactic anti-infection program consisting of piperacillin tazobactam 4.5 g, Q8 h + teicoplanin 0.4 g, QD + fluconazole 0.2 g, QD. The application times for the immunosuppressive drugs were tacrolimus (9:00, 21:00 oral administration) + mycophenolate mofetil (MMF, 10:00, 22:00 oral administration) + methylprednisolone (3 mg/kg/day, administered in four doses). The tacrolimus regimen was initiated with an initial dose of 0.03 mg/kg q12h, followed by continuous monitoring of tacrolimus blood concentrations (trough concentrations), during which time the patient’s dosage was adjusted according to the target blood concentration of 5–10 ng/mL, which was generally achieved in about 1 week. MMF was given as the body surface area × 0.3 every 12 h doses in each patient. The exclusion criteria were combined organ transplantation or secondary organ transplantation; missing *CYP3A5* gene detection data; intraoperative blood loss ≥ 800 mL or the need for fluid resuscitation (fluid volume ≥ 30 mL/kg/h); and patients who received splenectomy or a platelet transfusion before enrollment. Our study was conducted in accordance with the guidelines of the Declaration of Helsinki regarding ethical principles for research involving human subjects. The informed consent of the included patients and the acceptance of the study protocol by the Ethics Committee of our hospital (approval number: 22193-0-01) were obtained prior to the commencement of the study.

### 2.2. Data Collection and Grouping

Baseline characteristics (age, gender and BMI,), the drug metabolism enzyme *CYP3A5* data of the donors and recipients, laboratory data (evaluation of liver function, serum creatinine and platelet count) were recorded preoperatively and on PODs 1–10 and POD 28. Patients were divided into normal platelet (platelet count ≥ 100 × 10^9^/L) and thrombocytopenia (platelet count < 100 × 10^9^/L) [22] groups according to the platelet count on POD3. Tacrolimus dosage (mg/day) and blood concentrations were recorded on PODs 3, 7 and 28. The complications including infection, graft rejection, biliary leak, thrombosis and other outcomes were monitored for 180 days after DDLT.

*CYP3A5* mutations (6986 A > G, rs776746) were analyzed via PCR and according to the *CYP3A5* genotyping, divided into (donor/recipient) A*/A*, A*/GG, GG/A* and GG/GG groups [18]. The A*/A* type was associated with fast, the A*/GG, GG/A* types with intermediate and the GG/GG type with slow tacrolimus metabolism [23]. HLA genotyping was not performed since it is not routinely used in clinical practice [24].

### 2.3. Statistical Analysis

The data were analyzed using IBM SPSS Statistics for Windows (ver. 22.0, IBM Corp., Armonk, NY, USA). The normality of distributions was verified using the Kolmogorov–Smirnov test. Means ± standard deviations were calculated for continuous variables or medians (25th and 75th percentiles) were calculated if not normally distributed. Categorical variables, such as gender and etiology, were calculated as percentages (%). Comparisons of multiple groups divided by *CYP3A5* genotypes were performed using a Kruskal–Wallis analysis. Platelet counts over time and *CYP3A5* genotypes were compared using the repeated measure analysis of variance (ANOVA) method. A one-way ANOVA was used to look for differences among the A*/A*, A*/GG, GG/A* and GG/GG groups during the time frame, and the least significant difference (LSD) test was used for a post hoc analysis. Comparisons of two groups divided by platelet count on POD3 (normal platelet vs. thrombocytopenia) were made using an independent *t*-test for normally distributed data and for a non-normal distribution, Mann–Whitney’s U test was employed. A receiver operating characteristic (ROC) curve was used to assess the accuracy of the variables for the prediction of thrombocytopenia on POD3. A multivariate logistic regression was used to calculate the odds ratios and 95% confidence intervals (CIs) for risk factors of thrombocytopenia on POD3. The level of significance was set at *p* < 0.05 for all tests.

## 3. Results

### 3.1. Baseline Characteristics of Enrolled Patients

A total of 100 patients who fulfilled the criteria were finally enrolled. Patients in the A*/A*(*n* = 22), A*/GG (*n* = 20), GG/A*(*n* = 31) and GG/GG (*n* = 27) groups showed no significant differences in age, gender, BMI, etiology of liver disease and preoperative platelet counts. Also, spleen sizes and portal vein thrombosis incidences were not significantly different (Table 1). Two patients had died by the 180-day follow-up (one patient in the A*/A* group died of pulmonary hemorrhage on POD 7 and one in the GG/GG group of graft rejection on POD 30). The complications of DDLT included infections, graft rejection, biliary leak, thromboembolism and hemorrhage, from which only the latter one occurred significantly more frequently in the GG/GG group. The detailed platelet count on the third POD of the six patients in the GG/GG group who developed hemorrhages showed that only one patient in this group had a normal platelet count of 100 × 10^9^/L, but all patients had INR values of 1.03 to 1.25 (Table S1).

### 3.2. Changes in Platelet Counts in the First Month after DDLT

**Figure 1:**
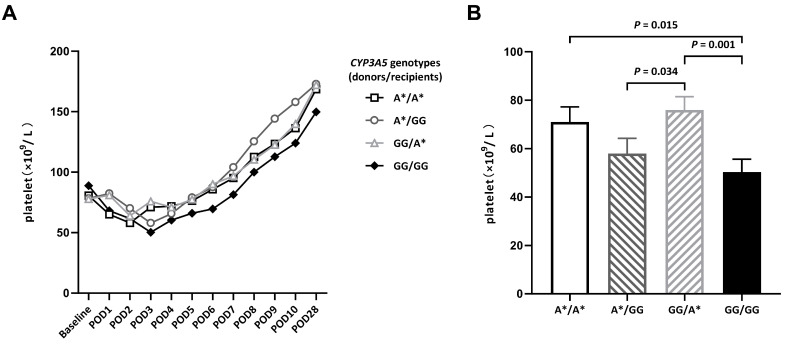
Platelet counts of patients with different *CYP3A5* genotypes after DDLT. (**A**) Platelet counts on indicated PODs after DDLT. (**B**) Platelet counts on POD3. POD: postoperative day.

Compared with preoperative values, platelet counts decreased and reached their lowest numbers on POD3 and continued to be low for a week. Patients divided by *CYP3A5* genotyping (donors/recipients) into the GG/GG group had the lowest platelet numbers in the follow-up period (Figure 1A). On POD 3, the platelet count in recipients with *CYP3A5* slow metabolism (A*/GG) was significantly lower than in donors (GG/A*) (Figure 1B). The reason for this result might be related to the slow metabolic rate of tacrolimus caused by the *CYP3A5* GG genotype of the recipients. The difference in the *CYP3A5* genotypes of the donors and recipients significantly affected the recipients’ pharmacokinetic parameters of tacrolimus after DDLT. In the GG/GG group, the tacrolimus dosage was the lowest, but these patients’ blood tacrolimus concentrations were higher than that of other groups. However, the effect disappeared on POD 28 (Table 2).

### 3.3. Correlation of Risk Factors for Thrombocytopenia

According to the platelet count on POD3 after DDLT, the patients were divided into two groups (normal platelet vs. thrombocytopenia). There were significant differences in spleen sizes (*p* = 0.000) and the blood concentrations of tacrolimus (*p* = 0.031) between the two groups (Table S2). However, after correction via multivariate logistic regression analysis, it was found that only the blood concentration of tacrolimus was a risk factor for developing thrombocytopenia after DDLT (Table 3). Blood concentrations of tacrolimus after DDLT were classified into three types (inadequate < 5 ng/mL; adequate 5–10 ng/mL; excessive > 10 ng/mL) according to the therapeutic target range of tacrolimus for liver transplantation. Compared with a blood concentration of tacrolimus < 5 ng/mL, the incidence of thrombocytopenia in the adequate group was 12.72 times higher than in the inadequate group. Seven patients were in the excessive group, and all of them developed thrombocytopenia.

**Figure 2:**
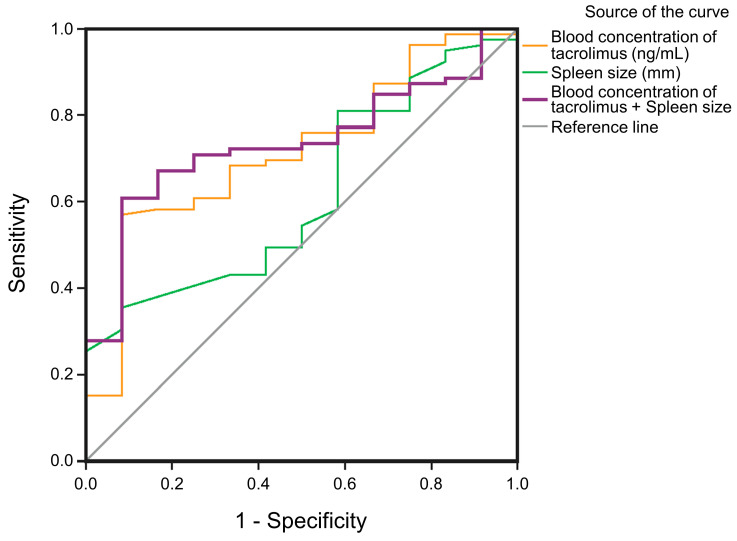
ROC curves for predicting thrombocytopenia on POD3.

AUCs for predicting thrombocytopenia on POD3 are shown for spleen sizes (mm) in combination with the blood concentration of tacrolimus on POD3 (ng/mL) AUC = 0.735 (purple line), spleen size (mm) AUC = 0.617 (green line) and a blood concentration of tacrolimus on POD3 (ng/mL) AUC = 0.719 (red line) (Figure 2). The blood concentrations of tacrolimus combined with spleen size on POD3 correlated most significantly with thrombocytopenia, with a sensitivity of 77.2% and a specificity of 41.7% (Table 4).

## 4. Discussion

The calcineurin inhibitor tacrolimus is used as a basic drug after liver and kidney transplantations due to its good anti-rejection actions, but it has a narrow therapeutic window because of nephrotoxicity, and its use makes patients prone to infectious disease [8]. In order to overcome these difficulties, tacrolimus doses are commonly adjusted via TDM [25]. However, TDM cannot provide adjustment information in the initial phase of treatment. (Table 2). Since the initial dosage of the drug was determined based on the patient’s weight (0.03 mg/kg, q12h), on POD3, there was no difference in drug dosages between fast-metabolizing patients (A*/A*) and slow-metabolizing patients (GG/GG). However, the drug concentration of tacrolimus in slow-metabolizing patients (GG/GG) was significantly higher than that in fast-metabolizing patients (A*/A*).

At the same time, compared to the other groups, platelet counts were the lowest in GG/GG patients whose blood concentrations of tacrolimus were the highest on POD3, and a correlation analysis revealed that the increase in the blood concentration of tacrolimus was significantly correlated with thrombocytopenia and hemorrhage. These data indicated that the *CYP3A5* genotype had some influence on the platelet count at least in the initial phase after DDLT, which, interestingly, was more pronounced when the recipient was a GG genotype bearer. In general, thrombocytopenia after liver transplantation is a common event with an incidence of 90% and a nadir at POD 3–5, with recovery normally occurring during the 2 weeks after transplantation, which was also the case in the present study [3,26]. The reasons for this post-operative thrombocytopenia are not clear but have been proposed to be the following:

1. Liver failure. As an important regulator of endogenous platelet production, thrombopoietin (TPO) is synthesized by the liver. Chronic liver diseases such as cirrhosis can lead to a reduction in TPO concentrations, which will affect megakaryocyte proliferation and differentiation and eventually lead to thrombocytopenia [27].
2. Excessive platelet consumption caused by massive blood loss during surgery. Combined organ transplantation and secondary organ transplantation may increase the degree of injury of patients, resulting in platelet consumption [28].
3. Fluid resuscitation may cause hemodilution, [29] but massive blood loss and secondary liver transplantations were excluded in this study.
4. Anticoagulation drugs can lead to a reduction in platelets [30].

However, in our research, using anticoagulation as a routine treatment after DDLT, there was no difference between the normal platelet and thrombocytopenia groups related to anticoagulation medication. Another reason might be platelet isolation caused by hypersplenism. This theory was first proposed by Aster in 1965 [31]. Through 51Cr labeled platelets, it was revealed that one-third of platelets are normally stored in the spleen in healthy individuals; in contrast, during liver cirrhosis with the enlargement of the spleen, the splenic blood pool is increased, and 50–90% of platelets are isolated in the spleen. Also, in the present study, the size of the spleen (cut-off value: 166.50 mm) was a risk factor for the degree of thrombocytopenia at POD3 which, in combination with the tacrolimus blood concentration (cut-off value: 4.74 ng/mL), led to an AUC of 0.735 with a specificity and sensitivity of 77.2% and 41.7%, respectively (Table 3).

However, the average platelet count reduction of 60% reported in the previous literature [26] was not reached in the present study since the average platelet count at baseline had already been essentially reduced and dropped on POD 3 by 12%, 26%, 3% and 43% in the A*/A*, A*/GG, GG/A* and GG/GG groups, respectively, compared to baseline. These values were closer to but still less than the 56.5 ± 23.5% reductions reported in a previous study [32]. There was no difference in the metabolizing ability for tacrolimus between the A*/GG and GG/A* groups of patients as intermediate metabolizers, although the concentration of tacrolimus appeared to be lower in patients in the GG/A* group than in those in the A*/GG group, but the apparent difference did not reach statistical significance. In terms of platelet counts, the effect of the tacrolimus concentration on platelet counts is further highlighted by the fact that patients in the GG/A* group had higher platelet numbers than those in the A*/GG group. Donor and acceptor double-slow-metabolism cases had a greater effect on the tacrolimus blood concentration and became the main research focus of the present study. However, the effect of genetic differences in donor and acceptor metabolism on the tacrolimus drug concentration is a topic of great interest and will be followed up on in future studies. Nevertheless, the present data indicate that higher tacrolimus serum concentrations, particularly in GG/GG patients, may have increased the general trend of platelet reduction.

One possibility is that tacrolimus may cause thrombotic microangiopathy (TMA). The incidence of tacrolimus-associated TMA has been reported to be approximately 1%, and TMA can occur at any stage of tacrolimus treatment after organ transplantation, being more common in the first year. Kaya et al. [33] conducted a retrospective analysis of 104 patients who were treated with tacrolimus after OLT from 1994 to 2010 and found that tacrolimus-related TMA occurred in 4 patients after transplantation. The mechanism of tacrolimus causing TMA may be that tacrolimus can cause endothelial cell damage, leading to the classical complement activation pathway [34]. Another possibility is that tacrolimus may cause thrombocytopenia due to its toxic effects on the hematopoietic system, but this side effect of tacrolimus is rare and has been documented in isolated case reports [35]. The related features include anemia and thrombotic thrombocytopenic purpura. There were four cases concerning red blood cell aplasia and only two cases of bone marrow inhibition (characterized by bone marrow hyperplasia, severe anemia, neutropenia, moderate thrombocytopenia and a decrease in megakaryocyte numbers). Complete recovery was achieved after the withdrawal of tacrolimus. De Simone et al. [36] conducted a study on patients treated with tacrolimus anti-rejection therapy after liver transplantation in which 11 patients treated with tacrolimus combined with MMF developed thrombocytopenia (6.2%) and 16 cases (8.9%) were in the tacrolimus combined with everolimus group, which might be explained by the fact that MMF had been used to treat immune thrombocytopenia in combination with a glucocorticoid [37]. However, since all patients in the present study received the same amount of MMF, this factor was not included in the covariates for the multivariate logistic regression analysis, though MMF may have contributed to reduced thrombocytopenia in some patients.

There were some limitations to the present study, including its retrospective design and a lack of TPO data, which are not included in routine clinical monitoring.

In conclusion, the valley blood concentration of tacrolimus ≥ 4.74 ng/mL on POD3 was a risk factor for elevating thrombocytopenia after DDLT. Recipients with the *CYP3A5* genotype GG/GG were more prone to exhibit thrombocytopenia and hemorrhage after DDLT. Initial tacrolimus concentrations may be adjusted after *CYP3A5* genotyping in patients admitted for liver transplantation.

## Figures and Tables

**Table 1:** Baseline characteristics of the study population.

Variables	A*/A* (*n* = 22)	A*/GG (*n* = 20)	GG/A* (*n* = 31)	GG/GG (*n* = 27)	*p*-Value
Age (years)	54.09 ± 9.78	51.75 ± 10.28	51.71 ± 11.21	52.04 ± 12.22	0.875
Gender					0.665
Male (*n*, %)	17 (77.3%)	18 (90%)	24 (77.4%)	23 (85.2%)	
Female (*n*, %)	5 (22.7%)	2 (10%)	7 (22.6%)	4 (14.8%)	
BMI (kg/m^2^)	23.29 ± 3.74	23.13 ± 4.52	23.22 ± 5.95	23.42 ± 3.44	0.996
Etiology of liver disease					
HCC (*n*, %)	12 (54.5%)	12 (60.0%)	12 (38.7%)	10 (37.0%)	
HBV cirrhosis (*n*, %)	4 (18.2%)	5 (25%)	11 (35.5%)	8 (29.6%)	
Alcoholic cirrhosis (*n*, %)	2 (9.1%)	2 (10%)	5 (16.1%)	5 (18.5%)	
ALD (*n*, %)	3 (13.6%)	1 (5%)	2 (6.5%)	3 (11.1%)	
Other (*n*, %)	1 (4.5%)	0 (0.0%)	1 (3.2%)	1 (3.7%)	
Platelet (×10^9^/L)	80.53 ± 43.39	78.30 ± 53.81	77.81 ± 49.23	88.78 ± 62.59	0.888
WBC (×10^9^/L)	3.21 ± 0.55	3.68 ± 0.39	2.96 ± 1.07	3.15 ± 0.92	0.348
Hb (g/dL)	10.55 ± 1.88	11.10 ± 1.72	10.69 ± 1.28	9.98 ± 1.36	0.683
INR (IQR)	1.36 (0.92)	1.33 (0.61)	1.48 (0.57)	1.48 (0.62)	0.736
ALT (U/L)	68.74 ± 52.35	39.89 ± 47.51	60.03 ± 64.58	88.32 ± 21.54	0.635
AST (U/L)	87.44 ± 28.60	61.11 ± 27.60	82.63 ± 21.31	88.41 ± 25.33	0.875
TB (mg/dL)	7.46 ± 2.39	6.22 ± 2.07	12.59 ± 2.58	10.54 ± 2.29	0.307
Albumin (g/dL)	3.28 ± 0.52	3.69 ± 1.08	3.77 ± 0.62	3.33 ± 0.69	0.473
Creatinine (mg/dL)	1.08 ± 0.18	1.17 ± 0.38	0.95 ± 0.45	1.05 ± 0.39	0.413
Spleen size (mm)	173.36 ± 11.42	179.45 ± 10.08	173.32 ± 9.46	179.29 ± 11.84	0.055
PVT (*n*, %)	2 (9.1%)	1 (5.0%)	3 (9.7%)	2 (7.4%)	0.458
MELD (*n*, %)	14.23 ± 8.35	13.10 ± 7.39	16.58 ± 11.52	16.89 ± 10.94	0.051
Child–Pugh (IQR)	8 (3)	9 (3)	8 (2)	8 (3)	0.380
Complications in 180 days after DDLT					
Infection (*n*, %)	2 (9.1%)	0 (0.0%)	2 (6.5%)	6 (22.2%)	0.162
Graft rejection (*n*, %)	1 (4.5%)	0 (0.0%)	1 (3.2%)	1 (3.7%)	0.509
Biliary leak (*n*, %)	2 (9.1%)	3 (15.0%)	8 (25.8%)	7 (25.9%)	0.383
Thromboembolism (*n*, %)	1 (4.5%)	2 (10.0%)	3 (9.7%)	4 (14.8%)	0.394
Hemorrhage (*n*, %)	1 (4.5%)	1 (5.0%)	2 (6.5%)	6 (22.2%) *	0.011

* Detailed platelet counts on the 3rd POD of the 6 patients in the GG/GG group who developed hemorrhages are shown in Table S1. A Kruskal–Wallis analysis was used to compare the baseline characteristics before DDLT and complications during the 180 days after DDLT among groups. ALD, autoimmune liver diseases; ALT, alanine aminotransferase; AST, aspartate aminotransferase; BMI, body mass index; Hb, hemoglobin; HBV, hepatitis B; HCC, hepatocellular carcinoma; MELD, model for end stage liver disease; PVT, portal vein thrombosis; TB, total bilirubin; WBC, white blood cell.

**Table 2:** Differences in platelet counts, dosages and blood concentration of tacrolimus among groups divided by *CYP3A5* (donors/recipients) after DDLT.

	A*/A*	A*/GG	GG/A*	GG/GG	*p*-Value
Platelet count (×10^9^/L)					
POD3	71.00 ± 6.22	57.95 ± 6.21	75.90 ± 5.56	50.29 ± 5.44	0.006
POD7	95.23 ± 11.24	104.00 ± 14.52	96.77 ± 9.74	81.44 ± 9.69	0.547
POD28	168.55 ± 14.89	172.85 ± 18.73	172.03 ± 14.86	149.81 ± 12.34	0.656
Blood concentration of tacrolimus (ng/mL)					
POD3	3.22 ± 1.85	5.59 ± 3.35	4.53 ± 2.72	6.22 ± 2.93	0.003
POD7	3.29 ± 1.74	5.80 ± 2.55	5.18 ± 2.19	6.24 ± 2.93	0.000
POD28	9.02 ± 5.09	7.69 ± 2.96	7.70 ± 3.55	8.97 ± 3.77	0.455
Tacrolimus dosage (mg/d)					
POD3	2.77 ± 0.69	2.80 ± 0.83	3.00 ± 1.06	2.48 ± 1.34	0.313
POD7	3.68 ± 1.21	3.47 ± 1.16	4.16 ± 1.18	2.70 ± 1.46	0.000
POD28	4.57 ± 1.54	3.83 ± 1.69	4.43 ± 1.41	3.48 ± 1.76	0.056

**Table 3:** Multivariate logistic regression analysis of the risk factors for thrombocytopenia.

Risk Factors	β	SE	Wald Value	Odds Ratio	95% CI	*p*-Value
Spleen size (mm)						
>180 vs. 120–150	1.77	1.55	1.302	5.89	0.28–123.74	0.254
150–180 vs. 120–150	1.24	1.46	0.712	3.46	0.194–61.57	0.399
Blood concentration of tacrolimus (ng/mL)						
>10 vs. <5	20.62	0.00	0.00	99.81	89.81–169.88	0.000
5–10 vs. <5	2.54	1.17	4.73	12.72	1.29–125.86	0.030
CYP3A5 (donor/recipient) GG/GG	0.477	1.05	0.21	1.61	0.21–12.49	0.648
Use anticoagulation	0.18	0.05	0.05	0.84	0.18–3.85	0.817
Creatinine ≥ 1.0 mg/dL	1.09	0.85	1.65	2.98	0.56–15.75	0.198
Child–Pugh ≥ 7	0.58	0.78	0.66	0.56	0.14–2.28	0.418

**Table 4:** Performance of multivariable models for correlating factors with thrombocytopenia on POD3.

Variable	Spleen Size (mm)	Blood Concentration of Tacrolimus (ng/mL)	Spleen Size + Blood Concentration of Tacrolimus
AUC	0.617	0.719	0.735
Cut-off value	166.50	4.74	0.82 ^#^
Sensitivity	81.0%	57.0%	77.2%
Specificity	41.7%	91.7%	41.7%
*p*-value	0.195	0.015	0.009

^#^ Predicted probability of combination = −6.018 + 0.037 × spleen size (mm) + 0.331 × blood concentration of tacrolimus (ng/mL).

## Data Availability

The datasets used and/or analysed during the current study are available from the corresponding author upon reasonable request.

## Supplementary Materials

The following supporting information can be downloaded at https://www.mdpi.com/article/10.3390/biomedicines11113088/s1, Table S1: Platelet count on the 3rd POD of the 6 patients in the GG/GG group which developed hemorrhages; Table S2: A Comparison of risk factors between normal platelet and thrombocytopenia groups.

## References

[1] Mehta N. (2021). Liver Transplantation Criteria for Hepatocellular Carcinoma, Including Posttransplant Management. Clin. Liver Dis..

[2] Millson C., Considine A., Cramp M.E., Holt A., Hubscher S., Hutchinson J., Jones K., Leithead J., Masson S., Menon K. (2020). Adult liver transplantation: UK clinical guideline—Part 2: Surgery and post-operation. Frontline Gastroenterol..

[3] Takahashi K., Nagai S., Safwan M., Liang C., Ohkohchi N. (2018). Thrombocytopenia after liver transplantation: Should we care?. World J. Gastroenterol..

[4] Gwiasda J., Schrem H., Klempnauer J., Kaltenborn A. (2017). Identifying independent risk factors for graft loss after primary liver transplantation. Langenbecks Arch. Surg..

[5] Takahashi K., Nagai S., Putchakayala K.G., Safwan M., Gosho M., Li A.Y., Kane W.J., Singh P.L., Rizzari M.D., Collins K.M. (2017). Prediction of biliary anastomotic stricture after deceased donor liver transplantation: The impact of platelet counts—A retrospective study. Transpl. Int..

[6] Akamatsu N., Sugawara Y., Kanako J., Arita J., Sakamoto Y., Hasegawa K., Kokudo N. (2017). Low Platelet Counts and Prolonged Prothrombin Time Early After Operation Predict the 90 Days Morbidity and Mortality in Living-donor Liver Transplantation. Ann. Surg..

[7] Armstrong V.W., Oellerich M. (2001). New developments in the immunosuppressive drug monitoring of cyclosporine, tacrolimus, and azathioprine. Clin. Biochem..

[8] Andrews L.M., Li Y., De Winter B.C.M., Shi Y.Y., Baan C.C., Van Gelder T., Hesselink D.A. (2017). Pharmacokinetic considerations related to therapeutic drug monitoring of tacrolimus in kidney transplant patients. Expert. Opin. Drug. Metab. Toxicol..

[9] Kershner R.P., Fitzsimmons W.E. (1996). Relationship of FK506 whole blood concentrations and efficacy and toxicity after liver and kidney transplantation. Transplantation.

[10] Nelson J., Alvey N., Bowman L., Schulte J., Segovia Maria C., McDermott J., Te H.S., Kapila N., Levine D.J., Gottlieb R.L. (2022). Consensus recommendations for use of maintenance immunosuppression in solid organ transplantation: Endorsed by the American College of Clinical Pharmacy, American Society of Transplantation, and the International Society for Heart and Lung Transplantation. Pharmacother. J. Hum. Pharmacol. Drug Ther..

[11] Sam W.J., Tham L.S., Holmes M.J., Aw M., Quak S.H., Lee K.H., Lim S.G., Prabhakaran K., Chan S.Y., Ho P.C. (2006). Population pharmacokinetics of tacrolimus in whole blood and plasma in asian liver transplant patients. Clin. Pharmacokinet..

[12] Looy S., Verplancke T., Benoit D., Hoste E., Maele G., De Turck F., Decruyenaere J. (2007). A novel approach for prediction of tacrolimus blood concentration in liver transplantation patients in the intensive care unit through support vector regression. Crit. Care.

[13] Yin S., Song T., Jiang Y., Li X., Fan Y., Lin T. (2019). Tacrolimus Trough Level at the First Month May Predict Renal Transplantation Outcomes Among Living Chinese Kidney Transplant Patients: A Propensity Score-Matched Analysis. Ther. Drug. Monit..

[14] Yoshikawa N., Urata S., Yasuda K., Sekiya H., Hirabara Y., Okumura M., Ikeda R. (2020). Retrospective analysis of the correlation between tacrolimus concentrations measured in whole blood and variations of blood cell counts in patients undergoing allogeneic haematopoietic stem cell transplantation. Eur. J. Hosp. Pharm..

[15] Sikma M.A., van Maarseveen E.M., van de Graaf E.A., Kirkels J.H., Verhaar M.C., Donker D.W., Kesecioglu J., Meulenbelt J. (2015). Pharmacokinetics and Toxicity of Tacrolimus Early After Heart and Lung Transplantation. Am. J. Transplant..

[16] Bouamar R., Shuker N., Hesselink D.A., Weimar W., Ekberg H., Kaplan B., Bernasconi C., van Gelder T. (2013). Tacrolimus predose concentrations do not predict the risk of acute rejection after renal transplantation: A pooled analysis from three randomized-controlled clinical trials. Am. J. Transplant..

[17] Ihara H., Shinkuma D., Ichikawa Y., Nojima M., Nagano S., Ikoma F. (1995). Intra- and interindividual variation in the pharmacokinetics of tacrolimus (FK506) in kidney transplant recipients—Importance of trough level as a practical indicator. Int. J. Urol..

[18] Birdwell K.A., Decker B., Barbarino J.M., Peterson J.F., Stein C.M., Sadee W., Wang D., Vinks A.A., He Y., Swen J.J. (2015). Clinical Pharmacogenetics Implementation Consortium (CPIC) Guidelines for CYP3A5 Genotype and Tacrolimus Dosing. Clin. Pharmacol. Ther..

[19] Radhakrishnan A., Kuppusamy G., Ponnusankar S., Mutalik S. (2021). Towards next-generation personalization of tacrolimus treatment: A review on advanced diagnostic and therapeutic approaches. Pharmacogenomics.

[20] Eng H.S., Mohamed Z., Calne R., Lang C.C., Mohd M.A., Seet W.T., Tan S.Y. (2006). The influence of CYP3A gene polymorphisms on cyclosporine dose requirement in renal allograft recipients. Kidney Int..

[21] Sun S., Ren Q., Zheng G., Han T., Feng F. (2019). The relationship between metabolic enzyme genetic polymorphisms and anti-tuberculosis drug-induced hepatotoxicity. Int. J. Clin. Exp. Med..

[22] Nagrebetsky A., Al-Samkari H., Davis N.M., Kuter D.J., Wiener-Kronish J.P. (2019). Perioperative thrombocytopenia: Evidence, evaluation, and emerging therapies. Br. J. Anaesth..

[23] Hendijani F., Azarpira N., Kaviani M. (2018). Effect of CYP3A5*1 expression on tacrolimus required dose after liver transplantation: A systematic review and meta-analysis. Clin. Transplant..

[24] Navarro V., Herrine S., Katopes C., Colombe B., Spain C.V. (2006). The effect of HLA class I (A and B) and class II (DR) compatibility on liver transplantation outcomes: An analysis of the OPTN database. Liver Transpl..

[25] Capron A., Musuamba F., Latinne D., Mourad M., Lerut J., Haufroid V., Wallemacq P.E. (2009). Validation of a liquid chromatography-mass spectrometric assay for tacrolimus in peripheral blood mononuclear cells. Ther. Drug. Monit..

[26] McCaughan G.W., Herkes R., Powers B., Rickard K., Gallagher N.D., Thompson J.F., Sheil A.G. (1992). Thrombocytopenia post liver transplantation. Correlations with pre-operative platelet count, blood transfusion requirements, allograft function and outcome. J. Hepatol..

[27] Saab S., Brown R.S. (2019). Management of Thrombocytopenia in Patients with Chronic Liver Disease. Dig. Dis. Sci..

[28] Unal D., Senayli Y., Polat R., Spahn D.R., Toraman F., Alkis N. (2020). Peri-operative blood transfusion in elective major surgery: Incidence, indications and outcome—An observational multicentre study. Blood Transfus.

[29] Kurokawa T., Ohkohchi N. (2017). Platelets in liver disease, cancer and regeneration. World J. Gastroenterol..

[30] Assfalg V., Huser N. (2016). Heparin-induced thrombocytopenia in solid organ transplant recipients: The current scientific knowledge. World J. Transplant..

[31] Aster R.H. (1965). Splenic platelet pooling as a cause of “hypersplenic” thrombocytopenia. Trans. Assoc. Am. Phys..

[32] Chatzipetrou M.A., Tsaroucha A.K., Weppler D., Pappas P.A., Kenyon N.S., Nery J.R., Khan M.F., Kato T., Pinna A.D., O’Brien C. (1999). Thrombocytopenia after liver transplantation. Transplantation.

[33] Kaya Z., Egritas O., Dalgic B. (2018). Tacrolimus-Induced Autoimmune Hemolytic Anemia in a Previously Reported Child With History of Thrombocytopenia Following Liver Transplant. Exp. Clin. Transplant..

[34] Jodele S., Fukuda T., Vinks A., Mizuno K., Laskin B.L., Goebel J., Dixon B.P., Teusink A., Pluthero F.G., Lu L. (2014). Eculizumab therapy in children with severe hematopoietic stem cell transplantation-associated thrombotic microangiopathy. Biol. Blood Marrow Transpl..

[35] Nosari A., Marbello L., De Carlis L.G., De Gasperi A., Muti G., Mancini V., Morra E. (2004). Bone marrow hypoplasia complicating tacrolimus (FK506) therapy. Int. J. Hematol..

[36] De Simone P., Carrai P., Coletti L., Ghinolfi D., Petruccelli S., Precisi A., Campani D., Marchetti P., Filipponi F. (2018). Everolimus vs Mycophenolate Mofetil in Combination with Tacrolimus: A Propensity Score-matched Analysis in Liver Transplantation. Transplant. Proc..

[37] Bradbury C.A., Pell J., Hill Q., Bagot C., Cooper N., Ingram J., Breheny K., Kandiyali R., Rayment R., Evans G. (2021). Mycophenolate Mofetil for First-Line Treatment of Immune Thrombocytopenia. New Engl. J. Med..

