# Supplementary material for: Effects of CYP3A5 Genotypes on Thrombocytopenia in Liver Transplantation Patients Treated with Tacrolimus
Source: Biomedicines. 2023 Nov 17;11(11):3088. doi: 10.3390/biomedicines11113088 (PMC10669143; doi:10.3390/biomedicines11113088)
Supplement: Supplementary file 1 [file biomedicines-11-03088-s001.zip › biomedicines-2660798-supplementary.pdf]

Table S1. Platelet count on the 3rd POD of the 6 patients in the GG/GG group which developed hemorrhages.

Patient	1	2	3	4	5	6
Platelet (×10^9/L)	38	3	100	67	46	54
INR	1.21	1.03	1.11	1.13	1.22	1.25
Bleeding Sites	Abdominal Cavity	Abdominal Cavity	Wound	Digestive Tract	Abdominal Cavity	Brain

Table S2. A Comparison of risk factors between normal platelet and thrombocytopenia groups.

**Variables**	**Total (*n* = 100)**	**Normal platelets (*n* = 14)**	**Thrombocytopenia (*n* = 86)**	***p*-value**
Age (years)	52.33 ± 10.90	48.00 ± 13.03	53.03 ± 10.43	0.109#
Gender				0.700&
Male (*n*, %)	82 (82%)	7 (50%)	75 (87%)	
Female (*n*, %)	18 (18%)	7 (50%)	11 (13%)	
BMI (kg/m2)	23.27 ± 4.55	24.65 ± 3.08	23.05 ± 4.73	0.223#
Spleen size (mm)	176.17 ± 10.97	160.64 ± 6.82	178.69 ± 9.31	0.000#
Parameters on POD3				
ALT (U/L)	335.45 ± 38.34	211.34 ± 36.74	354.03 ± 43.55	0.228#
AST (U/L)	167.45 ± 26.77	93.39 ± 49.94	179.50 ± 30.54	0.261#
TB (mg/dL)	3.25 ± 4.31	2.90 ± 0.38	3.65 ± 2.05	0.711#
Creatinine (mg/dL)	0.97 ± 0.71	0.98 ± 0.73	0.94 ± 0.61	0.859#
Blood concentration of tacrolimus (ng/mL)	4.84 ± 2.91	4.57 ± 2.85	6.38 ± 2.89	0.031#
Tacrolimus dosage (mg/day)	2.77 ± 1.04	2.74 ± 1.06	2.92 ± 0.83	0.542#
MMF dosage (mg/day)	789.00 ± 35.34	789.11 ± 36.69	788.60 ± 26.64	0.996#
Anticoagulation (*n*, %)	78 (78%)	12 (85%)	66 (77%)	0.194&
Child-Pugh (IQR)	6 (3)	5 (2)	6 (3)	0.094&
CYP3A5 GG/GG (*n*, %)	27 (27%)	3 (21.4%)	24 (27.9%)	0.617&

ALT, alanine aminotransferase; AST, aspartate aminotransferase; BMI, body mass index; MMF, mycophenolate mofetil; TB, total bilirubin.

#Between-group comparison was made using an independent *t*-test.

&Between-group comparison was made using the Mann–Whitney U test.
